# Supplementary material for: Development of oats flour and bitter gourd fortified cookies: Effects on physicochemical, antioxidant, antimicrobial, and sensory attributes
Source: PLoS One. 2025 Jan 9;20(1):e0316575. doi: 10.1371/journal.pone.0316575 (PMC11717233; doi:10.1371/journal.pone.0316575)
Supplement: S1 Dataset — (DOCX) [file pone.0316575.s001.docx]

**Experiment Values**

**Table 2: Weight, thickness, diameter, spread ratio, baking loss, and p^H^ of developed cookies at different treatment**

**Weight**

| **Samples** | **Weight (gm)** | **Mean ± SD** | **P-values** |
| --- | --- | --- | --- |
| Control | 11.09 | 10.86 ± 1.51 | 0.589 |
| Control | 12.24 |  |  |
| Control | 9.25 |  |  |
| T1 | 10.53 | 9.91 ± 1.38 |  |
| T1 | 10.89 |  |  |
| T1 | 8.33 |  |  |
| T2 | 10.47 | 9.37 ± 1.15 |  |
| T2 | 8.17 |  |  |
| T2 | 9.49 |  |  |
| T3 | 12.43 | 10.84 ± 1.45 |  |
| T3 | 9.59 |  |  |
| T3 | 10.52 |  |  |

**Thickness**

| **Samples** | **Thickness (mm)** | **Mean ± SD** | **P-values** |
| --- | --- | --- | --- |
| Control | 13.80 | 13.54 ± 0.72 | 0.047 |
| Control | 14.10 |  |  |
| Control | 12.73 |  |  |
| T1 | 12.58 | 12.06 ± 0.60 |  |
| T1 | 12.20 |  |  |
| T1 | 11.40 |  |  |
| T2 | 13.55 | 12.44 ± 0.96 |  |
| T2 | 11.88 |  |  |
| T2 | 11.90 |  |  |
| T3 | 11.30 | 11.12 ± 0.46 |  |
| T3 | 11.48 |  |  |
| T3 | 10.60 |  |  |

**Diameter (mm)**

| **Samples** | **Diameter (mm)** | **Mean ± SD** | **P-values** |
| --- | --- | --- | --- |
| Control | 36.20 | 36.82 ± 3.13 | 0.110 |
| Control | 40.23 |  |  |
| Control | 34.05 |  |  |
| T1 | 39.25 | 38.56 ± 1.056 |  |
| T1 | 39.10 |  |  |
| T1 | 37.35 |  |  |
| T2 | 35.50 | 35.37 ± 1.38 |  |
| T2 | 33.93 |  |  |
| T2 | 36.68 |  |  |
| T3 | 44.30 | 41.08 ± 2.82 |  |
| T3 | 39.05 |  |  |
| T3 | 39.90 |  |  |

**Spread ratio**

| **Samples** | **Spread ratio** | **Mean ± SD** | **P-values** |
| --- | --- | --- | --- |
| Control | 2.62 | 2.72 ± 0.12 | 0.014 |
| Control | 2.85 |  |  |
| Control | 2.68 |  |  |
| T1 | 3.12 | 3.20 ± 0.078 |  |
| T1 | 3.20 |  |  |
| T1 | 3.28 |  |  |
| T2 | 2.62 | 2.85 ± 0.23 |  |
| T2 | 2.86 |  |  |
| T2 | 3.08 |  |  |
| T3 | 3.92 | 3.69 ± 0.26 |  |
| T3 | 3.40 |  |  |
| T3 | 3.76 |  |  |

**Baking loss**

| **Samples** | **Baking loss (g/100)** | **Mean ± SD** | **P-values** |
| --- | --- | --- | --- |
| Control | 19.93 | 20.67 ± 1.54 | 0.371 |
| Control | 19.63 |  |  |
| Control | 22.44 |  |  |
| T1 | 20.48 | 22.15 ± 2.20 |  |
| T1 | 21.32 |  |  |
| T1 | 24.65 |  |  |
| T2 | 21.75 | 23.77 ± 1.76 |  |
| T2 | 24.60 |  |  |
| T2 | 24.96 |  |  |
| T3 | 20.80 | 22.12 ± 1.15 |  |
| T3 | 22.64 |  |  |
| T3 | 22.93 |  |  |

**pH**

| **Samples** | **P^H^** | **Mean ± SD** | **P-values** |
| --- | --- | --- | --- |
| Control | 6.90 | 6.92 ± .015 | 0.029 |
| Control | 6.92 |  |  |
| Control | 6.93 |  |  |
| T1 | 6.85 | 6.83 ± .026 |  |
| T1 | 6.80 |  |  |
| T1 | 6.84 |  |  |
| T2 | 6.88 | 6.81 ± .010 |  |
| T2 | 6.89 |  |  |
| T2 | 6.87 |  |  |
| T3 | 6.85 | 6.84 ± .026 |  |
| T3 | 6.86 |  |  |
| T3 | 6.81 |  |  |

**Table 3: Color value of developed cookies at different treatment**

| **Samples** | **L*** | **Mean ± SD** | **P-values** | **a*** | **Mean ± SD** | **P-values** | **b*** | **Mean ± SD** | **P-values** |
| --- | --- | --- | --- | --- | --- | --- | --- | --- | --- |
| Control | 66.09 | 69.06 ± 3.73 | 0.012 | 1.17 | 1.69 ± .47 | 0.00 | 13.65 | 14.39 ± .644 | 0.00 |
| Control | 73.24 |  |  | 2.10 |  |  | 14.73 |  |  |
| Control | 67.84 |  |  | 1.80 |  |  | 14.80 |  |  |
| T1 | 66.94 | 68.80 ± 1.89 | 0.012 | 1.16 | 1.52 ± .37 | 0.00 | 21.67 | 21.04 ± .56 | 0.00 |
| T1 | 68.74 |  |  | 1.50 |  |  | 20.81 |  |  |
| T1 | 70.72 |  |  | 1.90 |  |  | 20.63 |  |  |
| T2 | 56.18 | 59.28 ± 2.70 | 0.012 | -4.35 | -4.58 ± .54 | 0.00 | 16.28 | 16.54 ± .808 | 0.00 |
| T2 | 61.15 |  |  | -5.20 |  |  | 17.45 |  |  |
| T2 | 60.52 |  |  | -4.20 |  |  | 15.90 |  |  |
| T3 | 56.05 | 58.51 ± 2.17 | 0.012 | -3.53 | -4.24 ± .65 | 0.00 | 24.49 | 24.02 ± .408 | 0.00 |
| T3 | 60.15 |  |  | -4.40 |  |  | 23.73 |  |  |
| T3 | 59.32 |  |  | -4.80 |  |  | 23.85 |  |  |

**Table 4: Proximate composition of developed cookies (%)**

**Moisture (%)**

| **Samples** | **Moisture (%)** | **Mean ± SD** | **P-values** |
| --- | --- | --- | --- |
| Control | 4.3 | 4.23 ± .115 | 0.107 |
| Control | 4.3 |  |  |
| Control | 4.1 |  |  |
| T1 | 4.1 | 4.53 ± .38 |  |
| T1 | 4.7 |  |  |
| T1 | 4.8 |  |  |
| T2 | 4.4 | 4.40 ± .30 |  |
| T2 | 4.7 |  |  |
| T2 | 4.1 |  |  |
| T3 | 4.6 | 4.70 ± .173 |  |
| T3 | 4.9 |  |  |
| T3 | 4.6 |  |  |

**Protein (%)**

| **Samples** | **Protein (%)** | **Mean ± SD** | **P-values** |
| --- | --- | --- | --- |
| Control | 7.32 | 7.32 ± .0252 | 0.00 |
| Control | 7.30 |  |  |
| Control | 7.35 |  |  |
| T1 | 7.36 | 7.36 ± .020 |  |
| T1 | 7.34 |  |  |
| T1 | 7.38 |  |  |
| T2 | 7.02 | 7.02 ± .003 |  |
| T2 | 7.02 |  |  |
| T2 | 7.02 |  |  |
| T3 | 7.04 | 7.06 ± .0153 |  |
| T3 | 7.07 |  |  |
| T3 | 7.06 |  |  |

**Fat (%)**

| **Samples** | **Fat (%)** | **Mean ± SD** | **P-values** |
| --- | --- | --- | --- |
| Control | 15.25 | 14.30 ± 1.514 | 0.340 |
| Control | 15.10 |  |  |
| Control | 12.56 |  |  |
| T1 | 15.31 | 15.09 ± .61 |  |
| T1 | 15.57 |  |  |
| T1 | 14.40 |  |  |
| T2 | 12.97 | 13.82 ± .92 |  |
| T2 | 14.80 |  |  |
| T2 | 13.70 |  |  |
| T3 | 14.50 | 13.62 ± 1.28 |  |
| T3 | 12.15 |  |  |
| T3 | 14.20 |  |  |

**Ash (%)**

| **Samples** | **Ash (%)** | **Mean ± SD** | **P-values** |
| --- | --- | --- | --- |
| Control | 1.00 | 1.17 ± 0.153 | 0.015 |
| Control | 1.20 |  |  |
| Control | 1.30 |  |  |
| T1 | 1.30 | 1.23 ± 0.115 |  |
| T1 | 1.30 |  |  |
| T1 | 1.10 |  |  |
| T2 | 1.30 | 1.50 ± 0.20 |  |
| T2 | 1.70 |  |  |
| T2 | 1.50 |  |  |
| T3 | 1.60 | 1.67 ± 0.058 |  |
| T3 | 1.70 |  |  |
| T3 | 1.70 |  |  |

**Carbohydrate (%)**

| **Samples** | **Carbohydrate (%)** | **Mean ± SD** | **P-values** |
| --- | --- | --- | --- |
| Control | 72.13 | 72.97 ± 1.49 | 0.395 |
| Control | 72.10 |  |  |
| Control | 74.69 |  |  |
| T1 | 71.93 | 71.78 ± .628 |  |
| T1 | 71.09 |  |  |
| T1 | 72.32 |  |  |
| T2 | 73.31 | 72.92 ± 1.005 |  |
| T2 | 71.78 |  |  |
| T2 | 73.68 |  |  |
| T3 | 72.26 | 72.96 ± 1.06 |  |
| T3 | 74.18 |  |  |
| T3 | 72.44 |  |  |

**Energy value (Kcal)**

| **Samples** | **Energy value (Kcal)** | **Mean ± SD** | **P-values** |
| --- | --- | --- | --- |
| Control | 455.05 | 449.91 ± 7.60 | 0.262 |
| Control | 453.50 |  |  |
| Control | 441.18 |  |  |
| T1 | 454.96 | 452.40 ± 3.51 |  |
| T1 | 453.85 |  |  |
| T1 | 448.40 |  |  |
| T2 | 438.06 | 444.18 ± 5.43 |  |
| T2 | 448.40 |  |  |
| T2 | 446.10 |  |  |
| T3 | 447.70 | 442.62 ± 7.23 |  |
| T3 | 434.36 |  |  |
| T3 | 445.80 |  |  |

**Crude fiber (%)**

| **Samples** | **Crude fiber (%)** | **Mean ± SD** | **P-values** |
| --- | --- | --- | --- |
| Control | 3.250 | 3.33 ± 0.11 | 0.00 |
| Control | 3.460 |  |  |
| Control | 3.270 |  |  |
| T1 | 4.200 | 4.04 ± 0.20 |  |
| T1 | 4.080 |  |  |
| T1 | 3.840 |  |  |
| T2 | 6.020 | 6.01 ± 0.20 |  |
| T2 | 6.210 |  |  |
| T2 | 5.800 |  |  |
| T3 | 7.850 | 8.02 ± 0.20 |  |
| T3 | 8.200 |  |  |
| T3 | 8.020 |  |  |

**Table 5: Mineral contents of developed cookies**

| **Samples** | **Na (mg/100g)** | **Mean ± SD** | **P-values** | **Ca (mg/100g)** | **Mean ± SD** | **P-values** | **Zn (mg/100g)** | **Mean ± SD** | **P-values** |
| --- | --- | --- | --- | --- | --- | --- | --- | --- | --- |
| Control | 7.7075 | 771 ± 0.03 | 0.00 | 4.3505 | 4.35 ± 0.006d | 0.00 | .5886 | 58.8 ± 0.03 | 0.00 |
| Control | 7.7080 |  |  | 4.3506 |  |  | .5880 |  |  |
| Control | 7.7075 |  |  | 4.3505 |  |  | .5885 |  |  |
| T1 | 7.5446 | 754 ± 0.03 |  | 5.9868 | 5.99 ± 0.006c |  | .5890 | 59.0 ± 0.06 |  |
| T1 | 7.5450 |  |  | 5.9869 |  |  | .5900 |  |  |
| T1 | 7.5445 |  |  | 5.9868 |  |  | .5900 |  |  |
| T2 | 7.6300 | 763 ± 1.53 |  | 8.7338 | 8.73 ± 0.006a |  | .6660 | 66.7 ± 0.06 |  |
| T2 | 7.6500 |  |  | 8.7337 |  |  | .6670 |  |  |
| T2 | 7.6200 |  |  | 8.7338 |  |  | .6670 |  |  |
| T3 | 7.8679 | 787 ± .006 |  | 8.0694 | 8.07 ± 0.006b |  | .6485 | 64.8 ± 0.025 |  |
| T3 | 7.8680 |  |  | 8.0695 |  |  | .6482 |  |  |
| T3 | 7.8680 |  |  | 8.0695 |  |  | .6487 |  |  |

**Table 6: Antimicrobial activity of developed cookies**

**Day- 01**

| **Samples** | **Day-01 log(cfu/ml)** | **Mean ± SD** | **P-values** |
| --- | --- | --- | --- |
| Control | 7.30 | 7.40 ±.175 | 0.232 |
| Control | 7.60 |  |  |
| Control | 7.30 |  |  |
| T1 | 7.60 | 7.46 ± .151 |  |
| T1 | 7.30 |  |  |
| T1 | 7.48 |  |  |
| T2 | 7.00 | 7.10 ± .175 |  |
| T2 | 7.00 |  |  |
| T2 | 7.30 |  |  |
| T3 | 7.00 | 7.26 ± .241 |  |
| T3 | 7.30 |  |  |
| T3 | 7.48 |  |  |

**Day- 25**

| **Samples** | **Day-25 log (cfu/ml)** | **Mean ± SD** | **P-values** |
| --- | --- | --- | --- |
| Control | 8.30 | 8.30 ± .128 | 0.132 |
| Control | 8.18 |  |  |
| Control | 8.43 |  |  |
| T1 | 8.18 | 8.21 ± .040 |  |
| T1 | 8.20 |  |  |
| T1 | 8.26 |  |  |
| T2 | 8.04 | 8.00 ± .053 |  |
| T2 | 8.08 |  |  |
| T2 | 8.15 |  |  |
| T3 | 8.15 | 8.16 ± .017 |  |
| T3 | 8.18 |  |  |
| T3 | 8.15 |  |  |

**Relation between Day-01 and Day-25 Anti-microbial Activity**

| **Days** | **Control** | | **T1** | | **T2** | | **T3** | |
| --- | --- | --- | --- | --- | --- | --- | --- | --- |
|  | **Mean ± SD** | **P-values** | **Mean ± SD** | **P-values** | **Mean ± SD** | **P-values** | **Mean ± SD** | **P-values** |
| **Day-01** | 7.40 ± 0.174 | 0.002 | 7.46 ± 0.151 | 0.001 | 7.10 ± 0.174 | 0.001 | 7.26 ± 0.241 | 0.003 |
| **Day-25** | 8.30 ± 0.128 |  | 8.21 ± 0.040 |  | 8.09 ± 0.053 |  | 8.16 ± 0.017 |  |

**Antimicrobial activity of developed cookies**

**Figure 2: DPPH free radical scavenging activity values on developed cookies**

| **Samples** | **DPPH value** | **Mean ± SD** | **P-values** |
| --- | --- | --- | --- |
| Control | 9.62441315 | 13.14 ± 3.08 | 0.00 |
| Control | 14.43661972 |  |  |
| Control | 15.37558685 |  |  |
| T1 | 26.76056338 | 22.42 ± 4.53 |  |
| T1 | 22.76995305 |  |  |
| T1 | 17.72300469 |  |  |
| T2 | 74.53051643 | 75.51 ± 1.065 |  |
| T2 | 75.35211268 |  |  |
| T2 | 76.64319249 |  |  |
| T3 | 61.38497653 | 54.69 ± 6.073 |  |
| T3 | 49.53051643 |  |  |
| T3 | 53.16901408 |  |  |

**Figure 3: TBARS values on developed cookies**

| **Samples** | **TBA value** | **Mean ± SD** | **P-values** |
| --- | --- | --- | --- |
| Control | 1.21880 | 1.33 ± 0.146 | 0.003 |
| Control | 1.49580 |  |  |
| Control | 1.27420 |  |  |
| T1 | .94180 | 0.088 ± 0.056 |  |
| T1 | .83100 |  |  |
| T1 | .87532 |  |  |
| T2 | .78668 | 0.779 ± 0.018 |  |
| T2 | .75898 |  |  |
| T2 | .79222 |  |  |
| T3 | .97504 | 0.968 ± 0.028 |  |
| T3 | .93626 |  |  |
| T3 | .99166 |  |  |

**Figure 4: Sensory attributes of developed cookies**

**Hardness**

| **Samples** | **Hardness** | **Mean ± SD** | **P-values** |
| --- | --- | --- | --- |
| Control | 2 | 3.90 ± 0.876 | 0.128 |
| Control | 4 |  |  |
| Control | 5 |  |  |
| Control | 5 |  |  |
| Control | 4 |  |  |
| Control | 4 |  |  |
| Control | 4 |  |  |
| Control | 4 |  |  |
| Control | 3 |  |  |
| Control | 4 |  |  |
| T1 | 3 | 4.00 ± 0.667 |  |
| T1 | 4 |  |  |
| T1 | 5 |  |  |
| T1 | 5 |  |  |
| T1 | 4 |  |  |
| T1 | 3 |  |  |
| T1 | 4 |  |  |
| T1 | 4 |  |  |
| T1 | 4 |  |  |
| T1 | 4 |  |  |
| T2 | 2 | 3.50 ± 0.707 |  |
| T2 | 4 |  |  |
| T2 | 3 |  |  |
| T2 | 3 |  |  |
| T2 | 4 |  |  |
| T2 | 4 |  |  |
| T2 | 4 |  |  |
| T2 | 4 |  |  |
| T2 | 4 |  |  |
| T2 | 3 |  |  |
| T3 | 3 | 4.30 ± 0.675 |  |
| T3 | 5 |  |  |
| T3 | 5 |  |  |
| T3 | 5 |  |  |
| T3 | 5 |  |  |
| T3 | 4 |  |  |
| T3 | 4 |  |  |
| T3 | 4 |  |  |
| T3 | 4 |  |  |
| T3 | 4 |  |  |

**Appearance**

| **Samples** | **Appearance** | **Mean ± SD** | **P-values** |
| --- | --- | --- | --- |
| Control | 5 | 4.90 ± 0.316 | 0.014 |
| Control | 5 |  |  |
| Control | 5 |  |  |
| Control | 4 |  |  |
| Control | 5 |  |  |
| Control | 5 |  |  |
| Control | 5 |  |  |
| Control | 5 |  |  |
| Control | 5 |  |  |
| Control | 5 |  |  |
| T1 | 4 | 4.50 ± 0.707 |  |
| T1 | 5 |  |  |
| T1 | 5 |  |  |
| T1 | 3 |  |  |
| T1 | 4 |  |  |
| T1 | 4 |  |  |
| T1 | 5 |  |  |
| T1 | 5 |  |  |
| T1 | 5 |  |  |
| T1 | 5 |  |  |
| T2 | 2 | 3.90 ± 0.876 |  |
| T2 | 4 |  |  |
| T2 | 4 |  |  |
| T2 | 3 |  |  |
| T2 | 5 |  |  |
| T2 | 4 |  |  |
| T2 | 4 |  |  |
| T2 | 5 |  |  |
| T2 | 4 |  |  |
| T2 | 4 |  |  |
| T3 | 4 | 4.40 ± 0.699 |  |
| T3 | 5 |  |  |
| T3 | 4 |  |  |
| T3 | 3 |  |  |
| T3 | 5 |  |  |
| T3 | 5 |  |  |
| T3 | 5 |  |  |
| T3 | 5 |  |  |
| T3 | 4 |  |  |
| T3 | 4 |  |  |

**Crispiness**

| **Samples** | **Crispiness** | **Mean ± SD** | **P-values** |
| --- | --- | --- | --- |
| Control | 4 | 4.50 ± 0.527 | 0.009 |
| Control | 5 |  |  |
| Control | 5 |  |  |
| Control | 4 |  |  |
| Control | 5 |  |  |
| Control | 4 |  |  |
| Control | 4 |  |  |
| Control | 4 |  |  |
| Control | 5 |  |  |
| Control | 5 |  |  |
| T1 | 4 | 4.20 ± 0.632 |  |
| T1 | 5 |  |  |
| T1 | 5 |  |  |
| T1 | 4 |  |  |
| T1 | 4 |  |  |
| T1 | 3 |  |  |
| T1 | 4 |  |  |
| T1 | 4 |  |  |
| T1 | 5 |  |  |
| T1 | 4 |  |  |
| T2 | 3 | 3.60 ± 0.516 |  |
| T2 | 4 |  |  |
| T2 | 4 |  |  |
| T2 | 3 |  |  |
| T2 | 4 |  |  |
| T2 | 3 |  |  |
| T2 | 4 |  |  |
| T2 | 4 |  |  |
| T2 | 4 |  |  |
| T2 | 3 |  |  |
| T3 | 4 | 4.30 ± 0.675 |  |
| T3 | 5 |  |  |
| T3 | 4 |  |  |
| T3 | 4 |  |  |
| T3 | 5 |  |  |
| T3 | 5 |  |  |
| T3 | 5 |  |  |
| T3 | 4 |  |  |
| T3 | 4 |  |  |
| T3 | 3 |  |  |

**Flavor**

| **Samples** | **Flavor** | **Mean ± SD** | **P-values** |
| --- | --- | --- | --- |
| Control | 4 | 4.70 ± 0.483 | 0.078 |
| Control | 4 |  |  |
| Control | 5 |  |  |
| Control | 5 |  |  |
| Control | 5 |  |  |
| Control | 5 |  |  |
| Control | 5 |  |  |
| Control | 5 |  |  |
| Control | 5 |  |  |
| Control | 4 |  |  |
| T1 | 4 | 4.30 ± 0.675 |  |
| T1 | 4 |  |  |
| T1 | 5 |  |  |
| T1 | 3 |  |  |
| T1 | 4 |  |  |
| T1 | 4 |  |  |
| T1 | 5 |  |  |
| T1 | 5 |  |  |
| T1 | 4 |  |  |
| T1 | 5 |  |  |
| T2 | 2 | 3.80 ± 1.033 |  |
| T2 | 4 |  |  |
| T2 | 4 |  |  |
| T2 | 3 |  |  |
| T2 | 5 |  |  |
| T2 | 5 |  |  |
| T2 | 4 |  |  |
| T2 | 5 |  |  |
| T2 | 3 |  |  |
| T2 | 3 |  |  |
| T3 | 4 | 4.20 ± 0.632 |  |
| T3 | 4 |  |  |
| T3 | 4 |  |  |
| T3 | 3 |  |  |
| T3 | 5 |  |  |
| T3 | 4 |  |  |
| T3 | 5 |  |  |
| T3 | 5 |  |  |
| T3 | 4 |  |  |
| T3 | 4 |  |  |

**Overall Acceptability**

| **Samples** | **Overall Acceptability** | **Mean ± SD** | **P-values** |
| --- | --- | --- | --- |
| Control | 3 | 4.60 ± 0.699 | 0.010 |
| Control | 5 |  |  |
| Control | 4 |  |  |
| Control | 5 |  |  |
| Control | 5 |  |  |
| Control | 4 |  |  |
| Control | 5 |  |  |
| Control | 5 |  |  |
| Control | 5 |  |  |
| Control | 5 |  |  |
| T1 | 2 | 3.50 ± 0.972 |  |
| T1 | 2 |  |  |
| T1 | 5 |  |  |
| T1 | 3 |  |  |
| T1 | 3 |  |  |
| T1 | 4 |  |  |
| T1 | 4 |  |  |
| T1 | 4 |  |  |
| T1 | 4 |  |  |
| T1 | 4 |  |  |
| T2 | 2 | 3.40 ± 0.966 |  |
| T2 | 4 |  |  |
| T2 | 3 |  |  |
| T2 | 2 |  |  |
| T2 | 5 |  |  |
| T2 | 4 |  |  |
| T2 | 4 |  |  |
| T2 | 4 |  |  |
| T2 | 3 |  |  |
| T2 | 3 |  |  |
| T3 | 4 | 4.40 ± 0.699 |  |
| T3 | 5 |  |  |
| T3 | 5 |  |  |
| T3 | 3 |  |  |
| T3 | 5 |  |  |
| T3 | 4 |  |  |
| T3 | 5 |  |  |
| T3 | 5 |  |  |
| T3 | 4 |  |  |
| T3 | 4 |  |  |
