# Supplementary material for: Development of oats flour and bitter gourd fortified cookies: Effects on physicochemical, antioxidant, antimicrobial, and sensory attributes
Source: PLoS One. 2025 Jan 9;20(1):e0316575. doi: 10.1371/journal.pone.0316575 (PMC11717233; doi:10.1371/journal.pone.0316575)
Supplement: Attachment — Submitted filename: Response to Reviewers.docx [file pone.0316575.s002.docx]

**Response to Reviewers’**

**Reviewer #1**

**Comment:** Re-order key words alphabetically.

**Response:** Thank you for your comment. We’ve reordered the keywords alphabetically**.**

**Comment:** Delete "The primary constituents of cookies include carbohydrates, sugar, and fat, which can contribute to various health issues." in abstract.

**Response:** We’ve revised the abstract according to your guidelines.

**Comment:** In references, latin names should be italic. Some references should be renewed. There are lots of studies about cookies, therefore please cite papers published between 2020-2024.

**Response:** We’ve reviewed and reorganized the references according to your guidelines.

**Comment:** Table 7 should be given by a radar graph.

**Response:** We’ve created a radar graph to visualize the data of Table 7.

**Comment:** Please give the ethical permission number for sensory analysis.

**Response:** No animal or human study was involved in this work. For sensory evaluation, the participants (all above 16 years age) gave their consent to participate. Proper protocols approved by the ethical committee were followed during the sensory evaluation. Further the research was carried out following the guidelines provides by the Nutrition and Food Technology departmental ethics committee (Dr. Omar Faruque, Dr. Md. Alauddin and Dr. Md. Mahmudul Hasan) at Jashore University of Science and Technology, Jashore-7408, Bangladesh.

**Comment:** What is the originality of your work? There are lots of enrichment papers available in literature.

**Response:** The combination of oats flour and bitter gourd powder introduces a novel way to improve the nutritional profile of cookies, targeting health-conscious consumers and those with specific dietary needs. Utilization bitter gourd powder, which has less commonly used in baking offered a potential health benefits from conventional cookies. Overall, our work provides a new perspective on ingredient substitution in baked goods, with a focus on enhancing both nutritional value and product differentiation.

**Comment:** Why did you choose bitter gourd? Long-term consumption of bitter gourd can have negative health effects. For example, excessive consumption of bitter gourd is associated with miscarriage, liver inflammation, and irregular heartbeat. Also, women who are breastfeeding should also not eat this fruit because some bad substances of bitter melon can be passed into breast milk. People with low blood pressure (or a history of low blood pressure): Bitter melon has the effect of lowering blood pressure thanks to its ingredients Charantin, Polypeptid-P and Vicine.

**Response:** Thank you for raising important points regrading the use of bitter gourd. We selected bitter gourd for its distinct taste and potential health benefits, such as its antioxidant properties and its role in blood sugar regulation. Bitter gourd can have specific effects on health, particularly with long term consumption or excessive consumption. The amount of bitter gourd powder (3%) used in our cookies is minimal and its helps mitigate the risk of adverse effects.

Top of Form

Bottom of Form

**Reviewer #2:** The authors investigated the development of oats flour and bitter gourd fortified cookies, specifically on the effects on physicochemical, antioxidant, antimicrobial, and sensory attributes. The findings were very promising, and amply discussed. However, after several readings of it, there are a few areas that would help improve the quality of the work.

**Comment:** a) In the introduction, you talked about flour, oats, individually, without mentioning the process of making cookies using flour. Please, try to squeeze this process in, and condense/shorten the other aspects. Also, support this with a schematic flow diagram that depicts the various stages of cookie making process.

**Response**: We’ve updated the introduction section based on your feedback.

**Comment:** b) Where is actual gap, why is this study relevant, please, strengthen this aspect at the last paragraph of the introduction.

**Response:** We’ve clarified the research gap and strengthened the aspect of our study at the last paragraph of the introduction section.

**Comment**: c) The materials and methods is very good, it might be helpful to start this section, that is 2.1, as "Schematic overview of the experimental program", which comprise 4 sentences, first to introduce the flow diagram depicting the key stages of the work, sentence 2 and 3 connecting the objective of this work with the flow diagram, and sentence 4, reiterating that the lab procedures followed appropriate standards, and chemicals used for the analysis were at analytical grade.

Response: We’ve reviewed and reorganized the materials and methods part according to your guidelines.

**Comment:** d) After careful check of results and discussion, the reviewer would encourage authors to do more of discussion, deepen the the why? and how? and reduce the what? ok . Apply your discretion on this, at each section, tell us more about why this happened, and how it is related to existing literature, and if no literature has shown it, how it scientifically happens to be.

**Response:** We’ve revised the results and discussion based on your guidelines.

**Reviewer #3:**

**Comment:** The manuscript includes novelity and has contributed to scientific knowledge, however there is need to add more crisp in language of the manuscript. It needs to be improved without compromising the scientific intent. There is a need to add more recent and relevant references.

The methodology and discussion parts need to be more comprehensive to add interest of reader.
I appreciate the efforts of the authors and will be happy to see the changes as suggested.

**Response:** We’ve reviewed and addressed the methodology and discussion parts according to your guidelines.
